# Supplementary material for: Validity of self-reported hysterectomy: a prospective cohort study within the UK Collaborative Trial of Ovarian Cancer Screening (UKCTOCS)
Source: BMJ Open. 2014 Mar 3;4(3):e004421. doi: 10.1136/bmjopen-2013-004421 (PMC3939665; doi:10.1136/bmjopen-2013-004421)
Supplement: Author's manuscript [file bmjopen-2013-004421.draft_revisions.pdf]

VALIDITY OF SELF-REPORTED HYSTERECTOMY WITHIN THE UNITED KINGDOM COLLABORATIVE TRIAL OF OVARIAN CANCER SCREENING (UKCTOCS)

Journal:	*BMJ Open*
Manuscript ID:	bmjopen-2013-004421
Article Type:	Research
Date Submitted by the Author:	06-Nov-2013
Complete List of Authors:	Gentry-Maharaj, Aleksandra; Institute for Women's Health, University College London, Women's Cancer<br>Taylor, Henry; Institute for Women's Health, University College London, Women's Cancer<br>Kalsi, Jatinderpal; Institute for Women's Health, University College London, Women's Cancer<br>Ryan, Andy; Institute for Women's Health, University College London, Women's Cancer<br>Burnell, Matthew; Institute for Women's Health, University College London, Women's Cancer<br>Sharma, Aarti; Institute for Women's Health, University College London, Women's Cancer<br>Apostolidou, Sophia; Institute for Women's Health, University College London, Women's Cancer<br>Campbell, Stuart; Create Health Clinic,<br>Jacobs, Ian; Institute for Women's Health, University College London, Academic Health Science Centre<br>Menon, Usha; Institute for Women's Health, University College London, Women's Cancer
**Primary Subject Heading**:	Obstetrics and gynaecology
Secondary Subject Heading:	Oncology, Epidemiology
Keywords:	GYNAECOLOGY, EPIDEMIOLOGY, ONCOLOGY

SCHOLARONE™ Manuscripts

Research article

VALIDITY OF SELF-REPORTED HYSTERECTOMY - PROSPECTIVE COHORT STUDY WITHIN THE UNITED KINGDOM COLLABORATIVE TRIAL OF OVARIAN CANCER SCREENING (UKCTOCS)

Aleksandra Gentry-Maharaj[1], Henry Taylor[1], Jantiderpal Kalsi[1], Andy Ryan[1], Matthew Burnell[1], Aarti Sharma[1], Sophia Apostolidou[1], Stuart Campbell[2], Ian Jacobs [1,3], Usha Menon[1]

[1]Gynaecological Cancer Research Centre, Women's Cancer, UCL EGA Institute for Women's Health, London W1T 7DN, [2]Create Health Clinic, Harley Street, London W1G 6AJ, [3]Academic Health Science Centre, University of Manchester, Manchester M13 9NT

Corresponding author:

Dr Aleksandra Gentry-Maharaj

Gynaecological Cancer Research Centre

Women's Cancer

UCL EGA Institute for Women's Health

Maple House 1st Floor

149 Tottenham Court Road

London W1T 7DN

Tel: 020 3447 2118, Fax: 020 3447 2129; email: a.gentry-maharaj@ucl.ac.uk

Word count: 2,294

For peer review only - http://bmjopen.bmj.com/site/about/guidelines.xhtml

Abstract

Objective: To evaluate the validity of self-reported hysterectomy against the gold standard of uterine visualisation using pelvic ultrasound.

Design: Prospective cohort study.

Setting: United Kingdom Collaborative Trial of Ovarian Cancer Screening (UKCTOCS) based in 13 NHS Trusts in England, Wales and Northern Ireland.

Participants: Between April 2001 and October 2005, 48,215 postmenopausal women aged 50-74 randomised to the ultrasound screening arm of UKCTOCS underwent first (initial) scan on the trial.

Interventions: At recruitment, the women completed recruitment questionnaire (RQ) which included previous hysterectomy. The sonographer asked each woman regarding previous hysterectomy (interview-format, IF) prior to the scan. At the scan, in addition to ovarian morphology, endometrial-thickness (ET)/endometrial-abnormality were captured if uterus was visualised at the scan.

Outcome measures: Self-reported hysterectomy at RQ or IF was compared to ultrasound data on ET/endometrial abnormality (as surrogate uterine visualisation markers) on the first (initial) scan.

Results: Of 48,215 women, 3 had congenital uterine-agenesis and 218 inconclusive results. Uterus was visualised in 39,121 women. 8,871 self-reported hysterectomy at RQ, 8,641 at IF, 8,487 at both. Uterus was visualised in 39,123, 39,353, 38,969 in those not self-reporting hysterectomy at RQ, IF or both. Validity, sensitivity, specificity, positive predictive value (PPV) and negative predictive value (NPV) of using RQ alone, IF or both RQ/IF were 99.6%, 98.9%, 99.7%, 98.9% and 99.7%/98.9%, 98.4%, 99.1%, 95.9% and 99.7%, 99.8%, 99.6%, 99.9%, 99.4% and 99.9%, respectively.

Conclusions: Self-reported hysterectomy is highly accurate and valid source for studying long-term associations of hysterectomy with disease onset.

For peer review only

Trial registration: International Standard Randomised Controlled Trial Number (ISRCTN) – 22488978

Keywords: hysterectomy, self-reporting, ultrasound, validity, UKCTOCS

For peer review only

For peer review only - http://bmjopen.bmj.com/site/about/guidelines.xhtml

Strengths and limitations of the study

- In our study of 48,000 women, we show a very high validity of reporting of hysterectomy status by questionnaire or interview compared to ultrasound scan data
- This is the first report comparing self-reported hysterectomy status to the gold standard of ultrasound scanning
- It informs epidemiologists/clinical researchers that self-reported hysterectomy is a reliable data source when assessing its associations with disease risk
- The limitation of the study is that the scan form did not specifically capture whether uterus was seen or not and the reason

For peer review only

For peer review only - http://bmjopen.bmj.com/site/about/guidelines.xhtml

Introduction

Hysterectomy is one of the most commonly performed gynaecological operations.[1] Hysterectomy rates amongst post-menopausal women are 19%[2] to 25%[3] in the UK and up to 48% in the US.[4] Most women undergo hysterectomy for benign gynaecological conditions (uterine fibroids, excessive bleeding, endometriosis, uterine prolapse).[4,5] Over the last two decades an increasing body of literature has identified the impact of hysterectomy on disease risk. It has consistently been associated with reduced risk for ovarian cancer with a recent meta-analysis reporting a relative risk of ovarian cancer of 0.74 amongst women who had undergone hysterectomy.[6] Further associations with increased risk of cardiovascular disease[7] and reduced risk of breast cancer[8] have also been noted. In such studies, hysterectomy status is almost invariably ascertained by asking women whether they have had a hysterectomy or removal of the womb. However, there is a paucity of studies which analyse the accuracy of self-reporting of hysterectomy as a reliable method of data collection. We are aware of five small studies involving 79 to 455 women that have verified the validity of self-reported hysterectomy by review of medical notes or electronic records (Table 1).[9-13] Four of the five studies report agreement of 91% or over with one study of 452 women reporting validity of only 66%, as the hospital records could not confirm that the procedure had been undertaken.

Table 1: Studies comparing validity of self-reported hysterectomy compared to hospital/medical records

**Author, year**	**Study Design**	**Self-reported hysterectomy compared to source**	**No. reporting hysterectomy**	**No. who had hysterectomy status confirmed**	**No. where confirmation was impossible**	**Quoted validity of self report**
(Brett and Madans,	Cross sectional	Hospital Records	452	298	33	66%

For peer review only - http://bmjopen.bmj.com/site/about/guidelines.xhtml

1994)[12]						
(Green, Purdie et al., 1997)[10]	Case-control	Medical Records, surgeon's report, or GP	206	127	73	96%
(Phipps and Buist, 2009)[9]	Cross sectional	Surgical Pathology report (10%), imaging reports (35%) transcribed medical history (48%)	79	72	5	91%
(Colditz, Stampfer et al., 1987)[11]	Cross sectional	Medical records	212	199	12	99%
(Horwitz, 1986)[13]	Case control	Medical records	455	440	34	97%

In the ultrasound screening group of the UK Collaborative Trial of Ovarian Cancer Screening (UKCTOCS),[2] 50,639 women were randomised to an ultrasound scan of the pelvis following completion of a baseline questionnaire which included a question on hysterectomy. In this study, we report on the agreement between self-reported hysterectomy and ultrasound confirmation of presence/absence of a uterus.

Methods

Study design and subjects

UKCTOCS is a randomised controlled trial designed to assess whether screening for ovarian cancer has an impact on mortality from the disease. Between April 2001 and October 2005, 202,638 apparently healthy women aged 50 to 74, were recruited to the trial through 13 regional trial centres located in NHS Trusts in England, Wales and Northern Ireland. Women

For peer review only - http://bmjopen.bmj.com/site/about/guidelines.xhtml

completed a baseline questionnaire on lifestyle and medical history, which included a question on hysterectomy '(Have you ever had a hysterectomy (removal of the womb)?' (questionnaire format, RQ). The women were then randomised to screening either with (1) transvaginal ultrasound (Ultrasound group, USS; n=50,639) (2) CA125 interpreted by the Risk of Ovarian Cancer (ROC) algorithm (Multimodal group, MMS; n=50,640) or (3) control (no screening, n=101,359).[14]

The women randomised to the ultrasound arm of UKCTOCS were invited to attend for a transvaginal scan. At this visit, they were again questioned by the ultrasonographer about previous hysterectomy prior to the scan (interview format, IF). At each scan, in addition to data regarding the adnexae, the sonographer recorded the endometrial thickness (ET) and presence of any endometrial or uterine abnormalities. They also recorded any other relevant data as 'text' in the notes section.

Interpretation of the scan findings

The scan data was used to ascertain the presence or absence of a uterus. The uterus was deemed to be present if ET was measured or uterine abnormality e.g. 'fibroids' was noted. In addition, the uterus was considered to be present if the sonographer noted abnormal uterine position, presence of an intra-uterine device or made specific comments regarding uterine visualisation in the notes section. Uterus was deemed to be absent if there were no ET measurements nor any of the above were captured as text in the notes field. Scans with poor visualisation of the pelvis or where the procedure was abandoned due to discomfort were classified as 'inconclusive' and these women were excluded from the analysis.

Data analysis

Self-reported hysterectomy status on the questionnaire or at interview was compared to ultrasound findings of the presence or absence of a uterus.

For peer review only - http://bmjopen.bmj.com/site/about/guidelines.xhtml

We estimated the sensitivity as the proportion of women with an absent uterus confirmed on scan, who self-reported hysterectomy on RQ, at IF or both. Specificity was calculated as the proportion of women with a uterus confirmed on scan, who self-reported not having a hysterectomy. Positive Predictive value (PPV) was calculated as a proportion of women who did self-reported hysterectomy, where the scan confirmed absence of a uterus. Negative Predictive Value (NPV) was calculated as a proportion of women who did not self-report hysterectomy, where the scan confirmed presence of a uterus. Validity was calculated as a proportion of women who were accurate in their reporting (self-reported hysterectomy with uterus absent and those that did not report it with uterus present) out of all women.

We further evaluated whether age at randomisation or socio-economic status as derived from the Index of Multiple Deprivation (IMD) could affect the validity of reporting.

Results

Between 2001 and 2005, 50,639 women randomised to the ultrasound arm of UKCTOCS completed the recruitment questionnaire which included self-reported hysterectomy. Of them, 48,215 underwent the first (initial) scan on the trial. Of these women, 42,401 had a transvaginal (TVS), 4,325 a transabdominal (TA) scan and 1,489 had both performed at the same time. The median time from randomisation to scan was 2.7 weeks (interquartile range, IQR, 1.7-4.0). The uterus was reported to be present on the scan report in 38,221 and absent in 9,398 women. The latter included 3 women who had congenital uterine agenesis. In 593 women the ultrasound report was inconclusive with regard to presence or absence of uterus on this initial scan. Of these women, 242 had a TVS, 299 had a TA and 52 had both scans performed. As the 593 women went on to have 2 to 11 annual scans as part of the trial, these and all the other scans they have had were then queried to ascertain presence or absence of a uterus. On subsequent scans, in 353 presence of a uterus was recorded, 22 had the absence of a uterus clearly stated and 218 remained inconclusive due to difficulty

For peer review only - http://bmjopen.bmj.com/site/about/guidelines.xhtml

visualising the pelvis (Figure 1). Excluding the latter and women with congenital uterine agenesis, 47,994 women were therefore eligible for the analysis with ultrasound confirming presence of a uterus in 39,121 and absence of a uterus in 8,873.

Of the 47,994 women eligible for analysis, 8,871 self-reported hysterectomy at recruitment (RQ) and this was later confirmed on scan for 8,771 women. Of the 39,123 women who did not report hysterectomy on RQ, 39,021 had a uterus visualised at scan. In 102 women who did not report hysterectomy, a uterus was not found despite good visualisation of the pelvis.

At the interview (IF) prior to the scan, of the 47,994 women, the ultrasonographer recorded that 8,641 reported hysterectomy and 8,505 had it confirmed by scan data. Of the 39,353 women who did not report hysterectomy on IF, 38,985 had a uterus visualised at scan. Of the 8,871 women who self-reported hysterectomy on the RQ, 8,487 also reported it when interviewed immediately prior to the scan (IF) and 8457 had it confirmed by scan data. From the 39,123 women who did not report hysterectomy at RQ, 38,969 did not report it at IF either and the uterus was seen in 38,969. Seventy of the 384 women who self-reported hysterectomy on RQ but not on IF had a uterus visualised, whilst in 106 of the 154 women who did not report hysterectomy at RQ but did on IF the uterus was present. 54 women had uterine absence noted at the scan despite not reporting having had a hysterectomy at either the RQ or IF.

<u>Performance characteristics</u>

The validity, sensitivity, specificity, positive predictive value (PPV) and negative predictive value (NPV) of using RQ alone were 99.6%, 98.9%, 99.7%, 98.9% and 99.7%. Using the interview data alone, these were 98.9%, 98.4%, 99.1%, 95.9% and 99.7%. A McNemar test for paired proportions showed that RQ had greater validity than IF (99.6% versus 98.9%; $p<0.0001$). Consistent self-reporting at both RQ and IF resulted in validity, sensitivity, specificity, PPV and NPV of 99.8%, 99.6%, 99.9%, 99.4% and 99.9%, respectively (Table 2).

For peer review only - http://bmjopen.bmj.com/site/about/guidelines.xhtml

Table 2: Validity, sensitivity, specificity, PPV and NPV of self-reporting hysterectomy compared to the gold standard of ultrasound scanning

	**Self-reported hysterectomy**		
	**Recruitment Questionnaire (RQ)**	**Interview format (IF)**	**RQ and IF**
**Validity**	99.6%	98.9%	99.8%
**Sensitivity**	98.9%	98.4%	99.6%
**Specificity**	99.7%	99.1%	99.9%
**PPV**	98.9%	95.9%	99.4%
**NPV**	99.7%	99.7%	99.9%

<ins>Correlation with age and Index of Multiple Deprivation (IMD) score</ins>

The women who accurately reported the presence or absence of uterus (whether on RQ, IF or at both RQ and IF) were younger (median of 60.5, IQR 56.0-66.0) compared to those who were inaccurate in their reporting (median of 63.5, IQR 56.6-69.2) for RQ, 61.9 (IQR 56.5-67.7) for IF, 64.6 (IQR 57.9-69.1) for RQ and IF (Figure 2a). A Mann-Whitney test of the difference in rankings was significant at the 1% level (p=0.0006). The IMD score in those accurately reporting the presence or absence of a uterus was 15.0 (IQR 8.9-26.3) compared to 17.6 (IQR 10.9-29.4) for RQ, 16.4 (IQR 9.6-26.9) for IF and 18.2 (IQR 9.0-28.3) for both RQ and IF in those who were inaccurate (Figure 2b). A Mann-Whitney test showed some evidence of a difference in IMD score rankings (p=0.0214).

Discussion

To our knowledge, this is the largest study to date to assess the validity of self-reported hysterectomy by comparing it to a gold-standard of a pelvic ultrasound scan performed at a median of 2.7 weeks following questionnaire reporting. Self-reporting of hysterectomy was

For peer review only - http://bmjopen.bmj.com/site/about/guidelines.xhtml

correct in 99.6% of cases indicating that self-reporting (as captured in epidemiological studies) is an accurate measure of hysterectomy status. The validity of capturing hysterectomy using the questionnaire or interview was similar (99.6% versus 98.9%) though statistically significant. As the data on hysterectomy collected in most studies is usually ascertained through self-reporting, we feel that the data presented here can inform epidemiologists about the reliability of self-reporting of this variable.

The strengths of our study are (1) the size, (2) prospective design and (3) the fact that UKCTOCS participants were recruited from 13 centres across England, Wales and Northern Ireland through invitation from population registries – they are therefore as representative of the general female population aged 50-74 as is possible in clinical research.[14] We have previously observed a 'healthy- volunteer effect' in the trial[15] so the women participating in UKCTOCS may be more health-conscious and aware of medical procedures than the general population therefore being highly accurate in reporting. All women randomised to the ultrasound arm of UKCTOCS who attended for the initial scan were included in the analysis. The scans were in majority transvaginal, performed within 3 weeks of self-reporting with all ultrasonographers using similar ultrasound machines and following a uniform protocol and data capture form to record findings.[2] Where there was discrepancy or ultrasound records were inconclusive, all serial scans undertaken in the women during screening were analysed. Furthermore two types of self-reporting (questionnaire versus interview format) were assessed and their validity compared.

In our study, nearly 48,000 women were included in analysis with prospective evaluation of hysterectomy. Previous studies have been retrospective with medical note review used to confirm hysterectomy status. Logistical issues have meant that these studies were relatively small. They frequently encountered difficulties in acquiring notes because they relied upon patients remembering details of hospitals where a procedure, which potentially occurred decades ago, was undertaken and there was often poor recording of events.[9,10] In the multi-

For peer review only

For peer review only - http://bmjopen.bmj.com/site/about/guidelines.xhtml

centre Study of Women Across the Nation (SWAN), of the 3,302 participants, 239 reported hysterectomies of which for only 165 (69.3%) women the procedure could be confirmed. The remaining 74 could not be reviewed due to consent issues, loss to follow up or unavailability of medical records.[16] In addition some studies have used self-reported hysterectomy transcribed in medical histories as part of validation.[9] This has resulted in agreement of self-reported hysterectomy with medical records ranging from 66-99%.[9-13] A methodology that avoids several of these difficulties is to identify a cohort of women who have undergone hysterectomy using electronic outpatient records and subsequently investigate their self-reported hysterectomy status. Phipps and Buist, using this approach found that of 1,935 women with confirmed hysterectomy, 1,757 self-reported correctly, giving a sensitivity of 91%.[9]

Our study showed validity of self-reported hysterectomy of 99% in the women from the general population, aged 50-74. These figures are similar to the 100% accuracy rates reported in a subgroup of 200 women, for whom medical records could be obtained, participating in the Nurses' Study.[11] However this cohort was composed entirely of women with a nursing background who are well aware of the procedure, making it difficult to extrapolate to the wider population.

A limitation of our study is that the sonographer was not asked to specifically capture the presence or absence of a uterus. Endometrial thickness or endometrial abnormality were the only uterine details that were recorded.

A small proportion of women reported hysterectomy at recruitment but failed to do so at the scan. The reason for this discrepancy may be that either the sonographer did not question the woman or the woman did not hear the question at the time of the scan.

For peer review only

The women who were accurate in their reporting were younger than those that were inaccurate in their reporting of presence or absence of uterus. One of the reasons for this may be due to the procedure being done many years, even decades previously.

In our study 1.2% of women (576 of 47,994) were inconsistent in their self-reporting of hysterectomy status between the recruitment questionnaire and the interview carried out at a median 2.7 weeks later. Studies examining agreement of self-reports report a similarly low rate of inconsistency but these were made several years apart.[17,18] One of the reasons for this may be that the women were more accurate in reporting on the questionnaire as they had more time to fill this in compared to those attending their first scan in a busy clinic. Our and previous data indicate that capturing data via interviewer-administered questionnaire is a valid method to capture self-reported medical history.

Conclusion

Our data indicates that self-reported hysterectomy, whether through questionnaire or interview, is highly accurate and is a reliable data source for studying long term associations of hysterectomy with disease onset.

For peer review only

Acknowledgements

We are particularly grateful to the women throughout the UK who are participating in the trial and to the entire medical, nursing, and administrative staff who work on UKCTOCS.

Contributors: UM and AGM were involved in study design and concept and drafted the manuscript. UM, AGM and HT did the literature search for this manuscript and prepared the tables. UM, AGM and MB did the statistical analysis. JK, AR, AS, SA, SC and IJ were involved in the interpretation of the data. All authors critically revised the manuscript and approved the final version. UM is the guarantor.

Funding: UKCTOCS was core funded by the Medical Research Council, Cancer Research UK, and the Department of Health with additional support from the Eve Appeal, Special Trustees of Bart's and the London, and Special Trustees of UCLH and supported by researchers at the National Institute for Health Research University College London Hospitals Biomedical Research Centre. The funding source or the sponsor had no role in data collection, data analysis, data interpretation, or writing of the report. The researchers are independent from the funders.

Competing interests: IJ has a consultancy arrangement with Becton Dickinson in the field of tumour markers and ovarian cancer, UM has a financial interest through UCL Business and Abcodia Ltd in the third party exploitation of clinical trials biobanks which have been developed through the research at UCL. None of the other authors have any conflict of interest; no other relationships or activities that could appear to have influenced the submitted work.

Patient consent: Obtained.

For peer review only

Ethics approval: The study was approved by the UK North West Multicentre Research Ethics Committees (North West MREC 00/8/34) with site specific approval from the local regional ethics committees and the Caldicott guardians (data controllers) of the primary care trusts.

Data sharing statement: No additional data are available.

For peer review only

References

1. Johnson N, Barlow D, Lethaby A, Tavender E, Curr E, Garry R. Surgical approach to hysterectomy for benign gynaecological disease. *The Cochrane database of systematic reviews.* 2006(2):CD003677.
2. Menon U, Gentry-Maharaj A, Hallett R, et al. Sensitivity and specificity of multimodal and ultrasound screening for ovarian cancer, and stage distribution of detected cancers: results of the prevalence screen of the UK Collaborative Trial of Ovarian Cancer Screening (UKCTOCS). *The lancet oncology.* Apr 2009;10(4):327-340.
3. The Million Women Study: design and characteristics of the study population. The Million Women Study Collaborative Group. *Breast cancer research : BCR.* 1999;1(1):73-80.
4. Garry R. The future of hysterectomy. *BJOG : an international journal of obstetrics and gynaecology.* Feb 2005;112(2):133-139.
5. Merrill RM, Layman AB, Oderda G, Asche C. Risk estimates of hysterectomy and selected conditions commonly treated with hysterectomy. *Annals of epidemiology.* Mar 2008;18(3):253-260.
6. Hankinson SE, Hunter DJ, Colditz GA, et al. Tubal ligation, hysterectomy, and risk of ovarian cancer. A prospective study. *JAMA : the journal of the American Medical Association.* Dec 15 1993;270(23):2813-2818.
7. Ingelsson E, Lundholm C, Johansson AL, Altman D. Hysterectomy and risk of cardiovascular disease: a population-based cohort study. *European heart journal.* Mar 2011;32(6):745-750.
8. Press DJ, Sullivan-Halley J, Ursin G, et al. Breast cancer risk and ovariectomy, hysterectomy, and tubal sterilization in the women's contraceptive and reproductive experiences study. *American journal of epidemiology.* Jan 1 2011;173(1):38-47.
9. Phipps AI, Buist DS. Validation of self-reported history of hysterectomy and oophorectomy among women in an integrated group practice setting. *Menopause.* May-Jun 2009;16(3):576-581.
10. Green A, Purdie D, Green L, Dick ML, Bain C, Siskind V. Validity of self-reported hysterectomy and tubal sterilisation. The Survey of Women's Health Study Group. *Australian and New Zealand journal of public health.* Jun 1997;21(3):337-340.
11. Colditz GA, Stampfer MJ, Willett WC, et al. Reproducibility and validity of self-reported menopausal status in a prospective cohort study. *American journal of epidemiology.* Aug 1987;126(2):319-325.
12. Brett KM, Madans JH. Hysterectomy use: the correspondence between self-reports and hospital records. *American journal of public health.* Oct 1994;84(10):1653-1655.
13. Horwitz RI. Comparison of epidemiologic data from multiple sources. *Journal of chronic diseases.* 1986;39(11):889-896.
14. Menon U, Gentry-Maharaj A, Ryan A, et al. Recruitment to multicentre trials--lessons from UKCTOCS: descriptive study. *Bmj.* 2008;337:a2079.
15. Burnell M, Gentry-Maharaj A, Ryan A, et al. Impact on mortality and cancer incidence rates of using random invitation from population registers for recruitment to trials. *Trials.* 2011;12:61.
16. Morelli SS, Lian Y, Schott LL, Weiss G. Qualifications of Physicians Performing Hysterectomy: The Study of Women's Health Across the Nation. *Reproductive sciences.* Dec 10 2009.
17. Lucas R, Azevedo A, Barros H. Self-reported data on reproductive variables were reliable among postmenopausal women. *Journal of clinical epidemiology.* Sep 2008;61(9):945-950.
18. Bosetti C, Tavani A, Negri E, Trichopoulos D, La Vecchia C. Reliability of data on medical conditions, menstrual and reproductive history provided by hospital controls. *Journal of clinical epidemiology.* Sep 2001;54(9):902-906.

For peer review only - http://bmjopen.bmj.com/site/about/guidelines.xhtml

Figure legends

Figure 1: UKCTOCS participants randomised to the ultrasound group who underwent the initial scan on the trial which confirmed presence or absence of a uterus

Figure 2: **(a)** Kernel density plots of age at randomisation and **(b)** Index of Multiple Deprivation (IMD) for those that were accurate in their reporting versus those that were inaccurate

For peer review only

For peer review only - http://bmjopen.bmj.com/site/about/guidelines.xhtml

Figure 1

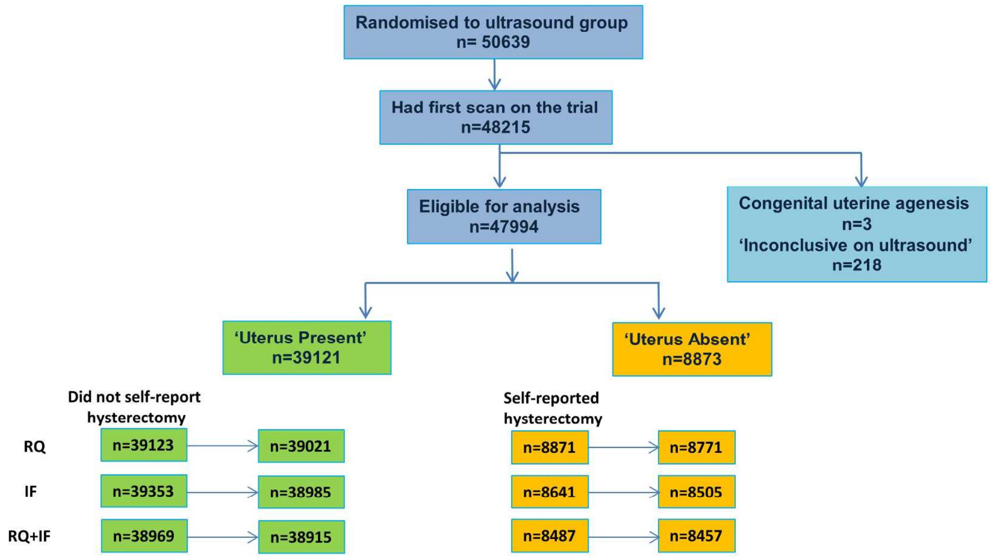

For peer review only - http://bmjopen.bmj.com/site/about/guidelines.xhtml

Figure 2

(a) (b)

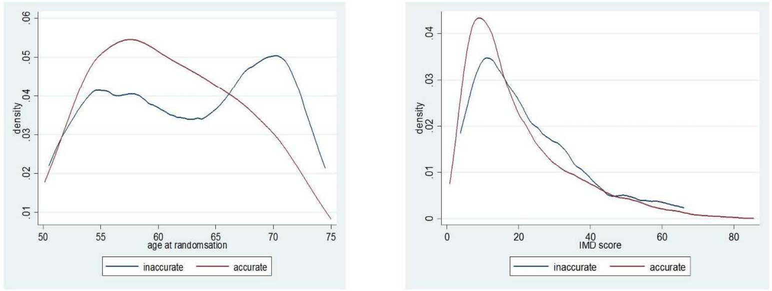

For peer review only

STROBE Statement		
**Item**	**Item No**	**Pages**
**Title and Abstract**	1	Pages 1-3
**Introduction**		
Background/rationale	2	Pages 5-6
Objectives	3	Page 6
**Methods**		
Study design	4	Pages 6-7
Setting	5	Pages 6-7
Participants	6	Pages 6-7
Variables	7	Pages 7-8
Data sources/measurement	8	Pages 7-8
Bias	9	Pages 7-8
Study size	10	Pages 6-8
Quantitative variables	11	Pages 7-8
Statistical methods	12	Pages 7-8
**Results**		
Participants	13	Pages 8-10
Descriptive data	14	Pages 8-10
Outcome data	15	Pages 8-10
Main results	16	Pages 8-10
Other analyses	17	Pages 8-10
**Discussion**		
Key results	18	Page 10-11
Limitations	19	Pages 12
Interpretation	20	Pages 11-12
Generalisability	21	Page 11-12
**Other information**		
Funding	22	Page 14

For peer review only

VALIDITY OF SELF-REPORTED HYSTERECTOMY - PROSPECTIVE COHORT STUDY WITHIN THE UNITED KINGDOM COLLABORATIVE TRIAL OF OVARIAN CANCER SCREENING (UKCTOCS)

Journal:	*BMJ Open*
Manuscript ID:	bmjopen-2013-004421.R1
Article Type:	Research
Date Submitted by the Author:	28-Jan-2014

Complete List of Authors:	Gentry-Maharaj, Aleksandra; Institute for Women's Health, University College London, Women's Cancer<br>Taylor, Henry; Institute for Women's Health, University College London, Women's Cancer<br>Kalsi, Jatinderpal; Institute for Women's Health, University College London, Women's Cancer<br>Ryan, Andy; Institute for Women's Health, University College London, Women's Cancer<br>Burnell, Matthew; Institute for Women's Health, University College London, Women's Cancer<br>Sharma, Aarti; Institute for Women's Health, University College London, Women's Cancer<br>Apostolidou, Sophia; Institute for Women's Health, University College London, Women's Cancer<br>Campbell, Stuart; Create Health Clinic,<br>Jacobs, Ian; Institute for Women's Health, University College London, Academic Health Science Centre<br>Menon, Usha; Institute for Women's Health, University College London, Women's Cancer

**Primary Subject Heading**:	Obstetrics and gynaecology
Secondary Subject Heading:	Oncology, Epidemiology
Keywords:	GYNAECOLOGY, EPIDEMIOLOGY, ONCOLOGY

SCHOLARONE™
Manuscripts

Research article

VALIDITY OF SELF-REPORTED HYSTERECTOMY - PROSPECTIVE COHORT STUDY WITHIN THE UNITED KINGDOM COLLABORATIVE TRIAL OF OVARIAN CANCER SCREENING (UKCTOCS)

Aleksandra Gentry-Maharaj[1], Henry Taylor[1], Jantiderpal Kalsi[1], Andy Ryan[1], Matthew Burnell[1], Aarti Sharma[1], Sophia Apostolidou[1], Stuart Campbell[2], Ian Jacobs [1,3], Usha Menon[1]

[1]Gynaecological Cancer Research Centre, Women's Cancer, UCL EGA Institute for Women's Health, London W1T 7DN, [2]Create Health Clinic, Harley Street, London W1G 6AJ, [3]Academic Health Science Centre, University of Manchester, Manchester M13 9NT

Corresponding author:

Dr Aleksandra Gentry-Maharaj

Gynaecological Cancer Research Centre

Women's Cancer

Institute for Women's Health

University College London

Maple House 1st Floor

149 Tottenham Court Road

London W1T 7DN

Tel: 020 3447 2118, Fax: 020 3447 2129; email: a.gentry-maharaj@ucl.ac.uk

Word count: 2,352

For peer review only

For peer review only - http://bmjopen.bmj.com/site/about/guidelines.xhtml

Abstract

Objective: To evaluate the validity of self-reported hysterectomy against the gold standard of uterine visualisation using pelvic ultrasound.

Design: Prospective cohort study.

Setting: United Kingdom Collaborative Trial of Ovarian Cancer Screening (UKCTOCS) based in 13 NHS Trusts in England, Wales and Northern Ireland.

Participants: Between April 2001 and October 2005, 48,215 postmenopausal women aged 50-74 randomised to the ultrasound screening arm of UKCTOCS underwent first (initial) scan on the trial.

Interventions: At recruitment, the women completed recruitment questionnaire (RQ) which included previous hysterectomy. The sonographer asked each woman regarding previous hysterectomy (interview-format, IF) prior to the scan. At the scan, in addition to ovarian morphology, endometrial-thickness (ET)/endometrial-abnormality were captured if uterus was visualised at the scan.

Outcome measures: Self-reported hysterectomy at RQ or IF was compared to ultrasound data on ET/endometrial abnormality (as surrogate uterine visualisation markers) on the first (initial) scan.

Results: Of 48,215 women, 3 had congenital uterine-agenesis and 218 inconclusive results. Uterus was visualised in 39,121 women. 8,871 self-reported hysterectomy at RQ, 8,641 at IF, 8,487 at both. Uterus was visualised in 39,123, 39,353, 38,969 in those not self-reporting hysterectomy at RQ, IF or both. Validity, sensitivity, specificity, positive predictive value (PPV) and negative predictive value (NPV) of using RQ alone, IF or both RQ/IF were 99.6%, 98.9%, 99.7%, 98.9% and 99.7%/98.9%, 98.4%, 99.1%, 95.9% and 99.7%, 99.8%, 99.6%, 99.9%, 99.4% and 99.9%, respectively.

Conclusions: Self-reported hysterectomy is highly accurate and valid source for studying long-term associations of hysterectomy with disease onset.

For peer review only - http://bmjopen.bmj.com/site/about/guidelines.xhtml

Trial registration: International Standard Randomised Controlled Trial Number (ISRCTN) – 22488978

Keywords: hysterectomy, self-reporting, ultrasound, validity, UKCTOCS

For peer review only

For peer review only - http://bmjopen.bmj.com/site/about/guidelines.xhtml

Strengths and limitations of the study

- In our study of 48,000 women, we show a very high validity of reporting of hysterectomy status by questionnaire or interview compared to ultrasound scan data
- This is the first report comparing self-reported hysterectomy status to the gold standard of ultrasound scanning
- It informs epidemiologists/clinical researchers that self-reported hysterectomy is a reliable data source when assessing its associations with disease risk
- The limitation of the study is that the scan form did not specifically capture whether uterus was seen or not and the reason

For peer review only

Introduction

Hysterectomy is one of the most commonly performed gynaecological operations.[1] Hysterectomy rates amongst post-menopausal women are 19%[2] to 25%[3] in the UK and up to 48% in the US.[4] Most women undergo hysterectomy for benign gynaecological conditions (uterine fibroids, excessive bleeding, endometriosis, uterine prolapse).[4,5] Over the last two decades an increasing body of literature has identified the impact of hysterectomy on disease risk. It has consistently been associated with reduced risk for ovarian cancer with a recent meta-analysis reporting a relative risk of ovarian cancer of 0.74 amongst women who had undergone hysterectomy.[6] Further associations with increased risk of cardiovascular disease[7] and reduced risk of breast cancer[8] have also been noted. In such studies, hysterectomy status is almost invariably ascertained by asking women whether they have had a hysterectomy or removal of the womb. However, there is a paucity of studies which analyse the accuracy of self-reporting of hysterectomy as a reliable method of data collection. We are aware of five small studies involving 79 to 455 women that have verified the validity of self-reported hysterectomy by review of medical notes or electronic records (Table 1).[9-13] Four of the five studies report agreement of 91% or over with one study of 452 women reporting validity of only 66%, as the hospital records could not confirm that the procedure had been undertaken.

Table 1: Studies comparing validity of self-reported hysterectomy compared to hospital/medical records

**Author, year**	**Study Design**	**Self-reported hysterectomy compared to source**	**No. reporting hysterectomy**	**No. who had hysterectomy status confirmed**	**No. where confirmation was impossible**	**Quoted validity of self report**
(Brett and Madans,	Cross sectional	Hospital Records	452	298	33	66%

For peer review only - http://bmjopen.bmj.com/site/about/guidelines.xhtml

1994)[12]						
(Green, Purdie et al., 1997)[10]	Case-control	Medical Records, surgeon's report, or GP	206	127	73	96%
(Phipps and Buist, 2009)[9]	Cross sectional	Surgical Pathology report (10%), imaging reports (35%) transcribed medical history (48%)	79	72	5	91%
(Colditz, Stampfer et al., 1987)[11]	Cross sectional	Medical records	212	199	12	99%
(Horwitz, 1986)[13]	Case control	Medical records	455	440	34	97%

In the ultrasound screening group of the UK Collaborative Trial of Ovarian Cancer Screening (UKCTOCS),[2] 50,639 women were randomised to an ultrasound scan of the pelvis following completion of a baseline questionnaire which included a question on hysterectomy. In this study, we report on the agreement between self-reported hysterectomy and ultrasound confirmation of presence/absence of a uterus.

Methods

Study design and subjects

UKCTOCS is a randomised controlled trial designed to assess whether screening for ovarian cancer has an impact on mortality from the disease. Between April 2001 and October 2005, 202,638 apparently healthy women aged 50 to 74, were recruited to the trial through 13 regional trial centres located in NHS Trusts in England, Wales and Northern Ireland. Women

For peer review only - http://bmjopen.bmj.com/site/about/guidelines.xhtml

completed a baseline questionnaire on lifestyle and medical history, which included a question on hysterectomy '(Have you ever had a hysterectomy (removal of the womb)?' (questionnaire format, RQ). The women were then randomised to screening either with (1) transvaginal ultrasound (Ultrasound group, USS; n=50,639) (2) CA125 interpreted by the Risk of Ovarian Cancer (ROC) algorithm (Multimodal group, MMS; n=50,640) or (3) control (no screening, n=101,359).[14]

The women randomised to the ultrasound arm of UKCTOCS were invited to attend for a transvaginal scan. At this visit, they were again questioned by the ultrasonographer about previous hysterectomy prior to the scan (interview format, IF). At each scan, in addition to data regarding the adnexae, the sonographer recorded the endometrial thickness (ET) and presence of any endometrial or uterine abnormalities. They also recorded any other relevant data as 'text' in the notes section.

Interpretation of the scan findings

The scan data was used to ascertain the presence or absence of a uterus. The uterus was deemed to be present if ET was measured or uterine abnormality e.g. 'fibroids' was noted. In addition, the uterus was considered to be present if the sonographer noted abnormal uterine position, presence of an intra-uterine device or made specific comments regarding uterine visualisation in the notes section. Uterus was deemed to be absent if there were no ET measurements nor any of the above were captured as text in the notes field. Scans with poor visualisation of the pelvis or where the procedure was abandoned due to discomfort were classified as 'inconclusive' and these women were excluded from the analysis.

Data analysis

Self-reported hysterectomy status on the questionnaire or at interview was compared to ultrasound findings of the presence or absence of a uterus.

For peer review only - http://bmjopen.bmj.com/site/about/guidelines.xhtml

We estimated the sensitivity as the proportion of women with an absent uterus confirmed on scan, who self-reported hysterectomy on RQ, at IF or both. Specificity was calculated as the proportion of women with a uterus confirmed on scan, who self-reported not having a hysterectomy. Positive Predictive value (PPV) was calculated as a proportion of women who did self-reported hysterectomy, where the scan confirmed absence of a uterus. Negative Predictive Value (NPV) was calculated as a proportion of women who did not self-report hysterectomy, where the scan confirmed presence of a uterus. Validity was calculated as a proportion of women who were accurate in their reporting (self-reported hysterectomy with uterus absent and those that did not report it with uterus present) out of all women.

We further evaluated whether age at randomisation or socio-economic status as derived from the Index of Multiple Deprivation (IMD) could affect the validity of reporting.

Results

Between 2001 and 2005, 50,639 women randomised to the ultrasound arm of UKCTOCS completed the recruitment questionnaire which included self-reported hysterectomy. Of them, 48,215 underwent the first (initial) scan on the trial. Of these women, 42,401 had a transvaginal (TVS), 4,325 a transabdominal (TA) scan and 1,489 had both performed at the same time. The median time from randomisation to scan was 2.7 weeks (interquartile range, IQR, 1.7-4.0). The uterus was reported to be present on the scan report in 38,221 and absent in 9,398 women. The latter included 3 women who had congenital uterine agenesis. In 593 women the ultrasound report was inconclusive with regard to presence or absence of uterus on this initial scan. Of these women, 242 had a TVS, 299 had a TA and 52 had both scans performed. As the 593 women went on to have 2 to 11 annual scans as part of the trial, these and all the other scans they have had were then queried to ascertain presence or absence of a uterus. On subsequent scans, in 353 presence of a uterus was recorded, 22 had the absence of a uterus clearly stated and 218 remained inconclusive due to difficulty

For peer review only - http://bmjopen.bmj.com/site/about/guidelines.xhtml

visualising the pelvis (Figure 1). Excluding the latter and women with congenital uterine agenesis, 47,994 women were therefore eligible for the analysis with ultrasound confirming presence of a uterus in 39,121 and absence of a uterus in 8,873.

Of the 47,994 women eligible for analysis, 8,871 self-reported hysterectomy at recruitment (RQ) and this was later confirmed on scan for 8,771 women. Of the 39,123 women who did not report hysterectomy on RQ, 39,021 had a uterus visualised at scan. In 102 women who did not report hysterectomy, a uterus was not found despite good visualisation of the pelvis.

At the interview (IF) prior to the scan, of the 47,994 women, the ultrasonographer recorded that 8,641 reported hysterectomy and 8,505 had it confirmed by scan data. Of the 39,353 women who did not report hysterectomy on IF, 38,985 had a uterus visualised at scan. Of the 8,871 women who self-reported hysterectomy on the RQ, 8,487 also reported it when interviewed immediately prior to the scan (IF) and 8457 had it confirmed by scan data. From the 39,123 women who did not report hysterectomy at RQ, 38,969 did not report it at IF either and the uterus was seen in 38,969. Seventy of the 384 women who self-reported hysterectomy on RQ but not on IF had a uterus visualised, whilst in 106 of the 154 women who did not report hysterectomy at RQ but did on IF the uterus was present. 54 women had uterine absence noted at the scan despite not reporting having had a hysterectomy at either the RQ or IF.

<u>Performance characteristics</u>

The validity, sensitivity, specificity, positive predictive value (PPV) and negative predictive value (NPV) of using RQ alone were 99.6%, 98.9%, 99.7%, 98.9% and 99.7%. Using the interview data alone, these were 98.9%, 98.4%, 99.1%, 95.9% and 99.7%. A McNemar test for paired proportions showed that RQ had greater validity than IF (99.6% versus 98.9%; $p<0.0001$). Consistent self-reporting at both RQ and IF resulted in validity, sensitivity, specificity, PPV and NPV of 99.8%, 99.6%, 99.9%, 99.4% and 99.9%, respectively (Table 2).

For peer review only - http://bmjopen.bmj.com/site/about/guidelines.xhtml

Table 2: Validity, sensitivity, specificity, PPV and NPV of self-reporting hysterectomy compared to the gold standard of ultrasound scanning

	**Self-reported hysterectomy**		
	**Recruitment Questionnaire (RQ)**	**Interview format (IF)**	**RQ and IF**
**Validity**	99.6%	98.9%	99.8%
**Sensitivity**	98.9%	98.4%	99.6%
**Specificity**	99.7%	99.1%	99.9%
**PPV**	98.9%	95.9%	99.4%
**NPV**	99.7%	99.7%	99.9%

Correlation with age and Index of Multiple Deprivation (IMD) score

The women who accurately reported the presence or absence of uterus (whether on RQ, IF or at both RQ and IF) were younger (median of 60.5, IQR 56.0-66.0) compared to those who were inaccurate in their reporting (median of 63.5, IQR 56.6-69.2) for RQ, 61.9 (IQR 56.5-67.7) for IF, 64.6 (IQR 57.9-69.1) for RQ and IF (Figure 2a). A Mann-Whitney test of the difference in rankings was significant at the 1% level (p=0.0006). The IMD score in those accurately reporting the presence or absence of a uterus was 15.0 (IQR 8.9-26.3) compared to 17.6 (IQR 10.9-29.4) for RQ, 16.4 (IQR 9.6-26.9) for IF and 18.2 (IQR 9.0-28.3) for both RQ and IF in those who were inaccurate (Figure 2b). A Mann-Whitney test showed some evidence of a difference in IMD score rankings (p=0.0214).

Discussion

To our knowledge, this is the largest study to date to assess the validity of self-reported hysterectomy by comparing it to a gold-standard of a pelvic ultrasound scan performed at a median of 2.7 weeks following questionnaire reporting. Self-reporting of hysterectomy was

For peer review only - http://bmjopen.bmj.com/site/about/guidelines.xhtml

correct in 99.6% of cases indicating that self-reporting (as captured in epidemiological studies) is an accurate measure of hysterectomy status. The validity of capturing hysterectomy using the questionnaire or interview was similar (99.6% versus 98.9%) though statistically significant. As the data on hysterectomy collected in most studies is usually ascertained through self-reporting, we feel that the data presented here can inform epidemiologists about the reliability of self-reporting of this variable.

The strengths of our study are (1) the size, (2) prospective design and (3) the fact that UKCTOCS participants were recruited from 13 centres across England, Wales and Northern Ireland through invitation from population registries – they are therefore as representative of the general female population aged 50-74 as is possible in clinical research.[14] We have previously observed a 'healthy- volunteer effect' in the trial[15] so the women participating in UKCTOCS may be more health-conscious and aware of medical procedures than the general population therefore being highly accurate in reporting. All women randomised to the ultrasound arm of UKCTOCS who attended for the initial scan were included in the analysis. The scans were in majority transvaginal, performed within 3 weeks of self-reporting with all ultrasonographers using similar ultrasound machines and following a uniform protocol and data capture form to record findings.[2] Where there was discrepancy or ultrasound records were inconclusive, all serial scans undertaken in the women during screening were analysed. Furthermore two types of self-reporting (questionnaire versus interview format) were assessed and their validity compared.

In our study, nearly 48,000 women were included in analysis with prospective evaluation of hysterectomy. Previous studies have been retrospective with medical note review used to confirm hysterectomy status. Logistical issues have meant that these studies were relatively small. They frequently encountered difficulties in acquiring notes because they relied upon patients remembering details of hospitals where a procedure, which potentially occurred decades ago, was undertaken and there was often poor recording of events.[9,10] In the multi-

For peer review only - http://bmjopen.bmj.com/site/about/guidelines.xhtml

For peer review only

centre Study of Women Across the Nation (SWAN), of the 3,302 participants, 239 reported hysterectomies of which for only 165 (69.3%) women the procedure could be confirmed. The remaining 74 could not be reviewed due to consent issues, loss to follow up or unavailability of medical records.[16] In addition some studies have used self-reported hysterectomy transcribed in medical histories as part of validation.[9] This has resulted in agreement of self-reported hysterectomy with medical records ranging from 66-99%.[9-13] A methodology that avoids several of these difficulties is to identify a cohort of women who have undergone hysterectomy using electronic outpatient records and subsequently investigate their self-reported hysterectomy status. Phipps and Buist, using this approach found that of 1,935 women with confirmed hysterectomy, 1,757 self-reported correctly, giving a sensitivity of 91%.[9]

Our study showed validity of self-reported hysterectomy of 99% in the UK women from the general population, aged 50-74. These figures are similar to the 100% accuracy rates reported in a subgroup of 200 women, for whom medical records could be obtained, participating in the Nurses' Study.[11] However this cohort was composed entirely of women with a nursing background who are well aware of the procedure, making it difficult to extrapolate to the wider population. Our results indicate that self-reporting of hysterectomy is highly accurate in women from the UK and we feel that our findings are applicable to women from other developed countries. However, it is possible that the validity of self-reporting of hysterectomy may be lower in those societies where there is a stigma attached to having undergone this procedure.

A limitation of our study is that the sonographer was not asked to specifically capture the presence or absence of a uterus. Endometrial thickness or endometrial abnormality were the only uterine details that were recorded.

For peer review only - http://bmjopen.bmj.com/site/about/guidelines.xhtml

A small proportion of women reported hysterectomy at recruitment but failed to do so at the scan. The reason for this discrepancy may be that either the sonographer did not question the woman or the woman did not hear the question at the time of the scan.

The women who were accurate in their reporting were younger than those that were inaccurate in their reporting of presence or absence of uterus. One of the reasons for this may be due to the procedure being done many years, even decades previously.

In our study 1.2% of women (576 of 47,994) were inconsistent in their self-reporting of hysterectomy status between the recruitment questionnaire and the interview carried out at a median 2.7 weeks later. Studies examining agreement of self-reports report a similarly low rate of inconsistency but these were made several years apart.[17,18] One of the reasons for this may be that the women were more accurate in reporting on the questionnaire as they had more time to fill this in compared to those attending their first scan in a busy clinic. Our and previous data indicate that capturing data via interviewer-administered questionnaire is a valid method to capture self-reported medical history.

Conclusion

Our data indicates that self-reported hysterectomy, whether through questionnaire or interview, is highly accurate and is a reliable data source for studying long term associations of hysterectomy with disease onset.

For peer review only - http://bmjopen.bmj.com/site/about/guidelines.xhtml

Acknowledgements

We are particularly grateful to the women throughout the UK who are participating in the trial and to the entire medical, nursing, and administrative staff who work on UKCTOCS.

Contributors: UM and AGM were involved in study design and concept and drafted the manuscript. UM, AGM and HT did the literature search for this manuscript and prepared the tables. UM, AGM and MB did the statistical analysis. JK, AR, AS, SA, SC and IJ were involved in the interpretation of the data. All authors critically revised the manuscript and approved the final version. UM is the guarantor.

Funding: UKCTOCS was core funded by the Medical Research Council, Cancer Research UK, and the Department of Health with additional support from the Eve Appeal, Special Trustees of Bart's and the London, and Special Trustees of UCLH and supported by researchers at the National Institute for Health Research University College London Hospitals Biomedical Research Centre. The funding source or the sponsor had no role in data collection, data analysis, data interpretation, or writing of the report. The researchers are independent from the funders.

Competing interests: IJ has a consultancy arrangement with Becton Dickinson in the field of tumour markers and ovarian cancer, UM has a financial interest through UCL Business and Abcodia Ltd in the third party exploitation of clinical trials biobanks which have been developed through the research at UCL. None of the other authors have any conflict of interest; no other relationships or activities that could appear to have influenced the submitted work.

Patient consent: Obtained.

For peer review only

Ethics approval: The study was approved by the UK North West Multicentre Research Ethics Committees (North West MREC 00/8/34) with site specific approval from the local regional ethics committees and the Caldicott guardians (data controllers) of the primary care trusts.

Data sharing statement: No additional data are available.

For peer review only

For peer review only - http://bmjopen.bmj.com/site/about/guidelines.xhtml

References

1. Johnson N, Barlow D, Lethaby A, et al. Surgical approach to hysterectomy for benign gynaecological disease. *The Cochrane database of systematic reviews.* 2006(2):CD003677.
2. Menon U, Gentry-Maharaj A, Hallett R, et al. Sensitivity and specificity of multimodal and ultrasound screening for ovarian cancer, and stage distribution of detected cancers: results of the prevalence screen of the UK Collaborative Trial of Ovarian Cancer Screening (UKCTOCS). *The lancet oncology.* Apr 2009;10(4):327-340.
3. The Million Women Study: design and characteristics of the study population. The Million Women Study Collaborative Group. *Breast cancer research : BCR.* 1999;1(1):73-80.
4. Garry R. The future of hysterectomy. *BJOG : an international journal of obstetrics and gynaecology.* Feb 2005;112(2):133-139.
5. Merrill RM, Layman AB, Oderda G, et al. Risk estimates of hysterectomy and selected conditions commonly treated with hysterectomy. *Annals of epidemiology.* Mar 2008;18(3):253-260.
6. Hankinson SE, Hunter DJ, Colditz GA, et al. Tubal ligation, hysterectomy, and risk of ovarian cancer. A prospective study. *JAMA : the journal of the American Medical Association.* Dec 15 1993;270(23):2813-2818.
7. Ingelsson E, Lundholm C, Johansson AL, Altman D. Hysterectomy and risk of cardiovascular disease: a population-based cohort study. *European heart journal.* Mar 2011;32(6):745-750.
8. Press DJ, Sullivan-Halley J, Ursin G, et al. Breast cancer risk and ovariectomy, hysterectomy, and tubal sterilization in the women's contraceptive and reproductive experiences study. *American journal of epidemiology.* Jan 1 2011;173(1):38-47.
9. Phipps AI, Buist DS. Validation of self-reported history of hysterectomy and oophorectomy among women in an integrated group practice setting. *Menopause.* May-Jun 2009;16(3):576-581.
10. Green A, Purdie D, Green L, et al. Validity of self-reported hysterectomy and tubal sterilisation. The Survey of Women's Health Study Group. *Australian and New Zealand journal of public health.* Jun 1997;21(3):337-340.
11. Colditz GA, Stampfer MJ, Willett WC, et al. Reproducibility and validity of self-reported menopausal status in a prospective cohort study. *American journal of epidemiology.* Aug 1987;126(2):319-325.
12. Brett KM, Madans JH. Hysterectomy use: the correspondence between self-reports and hospital records. *American journal of public health.* Oct 1994;84(10):1653-1655.
13. Horwitz RI. Comparison of epidemiologic data from multiple sources. *Journal of chronic diseases.* 1986;39(11):889-896.
14. Menon U, Gentry-Maharaj A, Ryan A, et al. Recruitment to multicentre trials--lessons from UKCTOCS: descriptive study. *Bmj.* 2008;337:a2079.
15. Burnell M, Gentry-Maharaj A, Ryan A, et al. Impact on mortality and cancer incidence rates of using random invitation from population registers for recruitment to trials. *Trials.* 2011;12:61.
16. Morelli SS, Lian Y, Schott LL, et al. Qualifications of Physicians Performing Hysterectomy: The Study of Women's Health Across the Nation. *Reproductive sciences.* Dec 10 2009.
17. Lucas R, Azevedo A, Barros H. Self-reported data on reproductive variables were reliable among postmenopausal women. *Journal of clinical epidemiology.* Sep 2008;61(9):945-950.
18. Bosetti C, Tavani A, Negri E, et al. Reliability of data on medical conditions, menstrual and reproductive history provided by hospital controls. *Journal of clinical epidemiology.* Sep 2001;54(9):902-906.

For peer review only - http://bmjopen.bmj.com/site/about/guidelines.xhtml

Figure legends

Figure 1: UKCTOCS participants randomised to the ultrasound group who underwent the initial scan on the trial which confirmed presence or absence of a uterus

Figure 2: **(a)** Kernel density plots of age at randomisation and **(b)** Index of Multiple Deprivation (IMD) for those that were accurate in their reporting versus those that were inaccurate

For peer review only

For peer review only - http://bmjopen.bmj.com/site/about/guidelines.xhtml

Research article

VALIDITY OF SELF-REPORTED HYSTERECTOMY - PROSPECTIVE COHORT STUDY WITHIN THE UNITED KINGDOM COLLABORATIVE TRIAL OF OVARIAN CANCER SCREENING (UKCTOCS)

For peer review only

Aleksandra Gentry-Maharaj[1], Henry Taylor[1], Jantiderpal Kalsi[1], Andy Ryan[1], Matthew Burnell[1], Aarti Sharma[1], Sophia Apostolidou[1], Stuart Campbell[2], Ian Jacobs [1,3], Usha Menon[1]

[1]Gynaecological Cancer Research Centre, Women's Cancer, UCL EGA Institute for Women's Health, London W1T 7DN, [2]Create Health Clinic, Harley Street, London W1G 6AJ, [3]Academic Health Science Centre, University of Manchester, Manchester M13 9NT

Corresponding author:

Dr Aleksandra Gentry-Maharaj

Gynaecological Cancer Research Centre

Women's Cancer

~~UCL EGA~~ Institute for Women's Health

University College London

Maple House 1st Floor

149 Tottenham Court Road

London W1T 7DN

Tel: 020 3447 2118, Fax: 020 3447 2129; email: a.gentry-maharaj@ucl.ac.uk

Word count: 2,352~~294~~

For peer review only - http://bmjopen.bmj.com/site/about/guidelines.xhtml

Abstract

Objective: To evaluate the validity of self-reported hysterectomy against the gold standard of uterine visualisation using pelvic ultrasound.

Design: Prospective cohort study.

Setting: United Kingdom Collaborative Trial of Ovarian Cancer Screening (UKCTOCS) based in 13 NHS Trusts in England, Wales and Northern Ireland.

Participants: Between April 2001 and October 2005, 48,215 postmenopausal women aged 50-74 randomised to the ultrasound screening arm of UKCTOCS underwent first (initial) scan on the trial.

Interventions: At recruitment, the women completed recruitment questionnaire (RQ) which included previous hysterectomy. The sonographer asked each woman regarding previous hysterectomy (interview-format, IF) prior to the scan. At the scan, in addition to ovarian morphology, endometrial-thickness (ET)/endometrial-abnormality were captured if uterus was visualised at the scan.

Outcome measures: Self-reported hysterectomy at RQ or IF was compared to ultrasound data on ET/endometrial abnormality (as surrogate uterine visualisation markers) on the first (initial) scan.

Results: Of 48,215 women, 3 had congenital uterine-agenesis and 218 inconclusive results. Uterus was visualised in 39,121 women. 8,871 self-reported hysterectomy at RQ, 8,641 at IF, 8,487 at both. Uterus was visualised in 39,123, 39,353, 38,969 in those not self-reporting hysterectomy at RQ, IF or both. Validity, sensitivity, specificity, positive predictive value (PPV) and negative predictive value (NPV) of using RQ alone, IF or both RQ/IF were 99.6%, 98.9%, 99.7%, 98.9% and 99.7%/98.9%, 98.4%, 99.1%, 95.9% and 99.7%, 99.8%, 99.6%, 99.9%, 99.4% and 99.9%, respectively.

Conclusions: Self-reported hysterectomy is highly accurate and valid source for studying long-term associations of hysterectomy with disease onset.

For peer review only - http://bmjopen.bmj.com/site/about/guidelines.xhtml

Trial registration: International Standard Randomised Controlled Trial Number (ISRCTN) – 22488978

Keywords: hysterectomy, self-reporting, ultrasound, validity, UKCTOCS

For peer review only

For peer review only - http://bmjopen.bmj.com/site/about/guidelines.xhtml

Strengths and limitations of the study

- In our study of 48,000 women, we show a very high validity of reporting of hysterectomy status by questionnaire or interview compared to ultrasound scan data
- This is the first report comparing self-reported hysterectomy status to the gold standard of ultrasound scanning
- It informs epidemiologists/clinical researchers that self-reported hysterectomy is a reliable data source when assessing its associations with disease risk
- The limitation of the study is that the scan form did not specifically capture whether uterus was seen or not and the reason

For peer review only

For peer review only - http://bmjopen.bmj.com/site/about/guidelines.xhtml

Introduction

Hysterectomy is one of the most commonly performed gynaecological operations.[1] Hysterectomy rates amongst post-menopausal women are 19%[2] to 25%[3] in the UK and up to 48% in the US.[4] Most women undergo hysterectomy for benign gynaecological conditions (uterine fibroids, excessive bleeding, endometriosis, uterine prolapse).[4,5] Over the last two decades an increasing body of literature has identified the impact of hysterectomy on disease risk. It has consistently been associated with reduced risk for ovarian cancer with a recent meta-analysis reporting a relative risk of ovarian cancer of 0.74 amongst women who had undergone hysterectomy.[6] Further associations with increased risk of cardiovascular disease[7] and reduced risk of breast cancer[8] have also been noted. In such studies, hysterectomy status is almost invariably ascertained by asking women whether they have had a hysterectomy or removal of the womb. However, there is a paucity of studies which analyse the accuracy of self-reporting of hysterectomy as a reliable method of data collection. We are aware of five small studies involving 79 to 455 women that have verified the validity of self-reported hysterectomy by review of medical notes or electronic records (Table 1).[9-13] Four of the five studies report agreement of 91% or over with one study of 452 women reporting validity of only 66%, as the hospital records could not confirm that the procedure had been undertaken.

Table 1: Studies comparing validity of self-reported hysterectomy compared to hospital/medical records

**Author, year**	**Study Design**	**Self-reported hysterectomy compared to source**	**No. reporting hysterectomy**	**No. who had hysterectomy status confirmed**	**No. where confirmation was impossible**	**Quoted validity of self report**
(Brett and Madans,	Cross sectional	Hospital Records	452	298	33	66%

For peer review only - http://bmjopen.bmj.com/site/about/guidelines.xhtml

1994)[12]						
(Green, Purdie et al., 1997)[10]	Case-control	Medical Records, surgeon's report, or GP	206	127	73	96%
(Phipps and Buist, 2009)[9]	Cross sectional	Surgical Pathology report (10%), imaging reports (35%) transcribed medical history (48%)	79	72	5	91%
(Colditz, Stampfer et al., 1987)[11]	Cross sectional	Medical records	212	199	12	99%
(Horwitz, 1986)[13]	Case control	Medical records	455	440	34	97%

In the ultrasound screening group of the UK Collaborative Trial of Ovarian Cancer Screening (UKCTOCS),[2] 50,639 women were randomised to an ultrasound scan of the pelvis following completion of a baseline questionnaire which included a question on hysterectomy. In this study, we report on the agreement between self-reported hysterectomy and ultrasound confirmation of presence/absence of a uterus.

Methods

Study design and subjects

UKCTOCS is a randomised controlled trial designed to assess whether screening for ovarian cancer has an impact on mortality from the disease. Between April 2001 and October 2005, 202,638 apparently healthy women aged 50 to 74, were recruited to the trial through 13 regional trial centres located in NHS Trusts in England, Wales and Northern Ireland. Women

For peer review only - http://bmjopen.bmj.com/site/about/guidelines.xhtml

completed a baseline questionnaire on lifestyle and medical history, which included a question on hysterectomy '(Have you ever had a hysterectomy (removal of the womb)?' (questionnaire format, RQ). The women were then randomised to screening either with (1) transvaginal ultrasound (Ultrasound group, USS; n=50,639) (2) CA125 interpreted by the Risk of Ovarian Cancer (ROC) algorithm (Multimodal group, MMS; n=50,640) or (3) control (no screening, n=101,359).[14]

The women randomised to the ultrasound arm of UKCTOCS were invited to attend for a transvaginal scan. At this visit, they were again questioned by the ultrasonographer about previous hysterectomy prior to the scan (interview format, IF). At each scan, in addition to data regarding the adnexae, the sonographer recorded the endometrial thickness (ET) and presence of any endometrial or uterine abnormalities. They also recorded any other relevant data as 'text' in the notes section.

Interpretation of the scan findings

The scan data was used to ascertain the presence or absence of a uterus. The uterus was deemed to be present if ET was measured or uterine abnormality e.g. 'fibroids' was noted. In addition, the uterus was considered to be present if the sonographer noted abnormal uterine position, presence of an intra-uterine device or made specific comments regarding uterine visualisation in the notes section. Uterus was deemed to be absent if there were no ET measurements nor any of the above were captured as text in the notes field. Scans with poor visualisation of the pelvis or where the procedure was abandoned due to discomfort were classified as 'inconclusive' and these women were excluded from the analysis.

Data analysis

Self-reported hysterectomy status on the questionnaire or at interview was compared to ultrasound findings of the presence or absence of a uterus.

For peer review only - http://bmjopen.bmj.com/site/about/guidelines.xhtml

We estimated the sensitivity as the proportion of women with an absent uterus confirmed on scan, who self-reported hysterectomy on RQ, at IF or both. Specificity was calculated as the proportion of women with a uterus confirmed on scan, who self-reported not having a hysterectomy. Positive Predictive value (PPV) was calculated as a proportion of women who did self-reported hysterectomy, where the scan confirmed absence of a uterus. Negative Predictive Value (NPV) was calculated as a proportion of women who did not self-report hysterectomy, where the scan confirmed presence of a uterus. Validity was calculated as a proportion of women who were accurate in their reporting (self-reported hysterectomy with uterus absent and those that did not report it with uterus present) out of all women.

We further evaluated whether age at randomisation or socio-economic status as derived from the Index of Multiple Deprivation (IMD) could affect the validity of reporting.

Results

Between 2001 and 2005, 50,639 women randomised to the ultrasound arm of UKCTOCS completed the recruitment questionnaire which included self-reported hysterectomy. Of them, 48,215 underwent the first (initial) scan on the trial. Of these women, 42,401 had a transvaginal (TVS), 4,325 a transabdominal (TA) scan and 1,489 had both performed at the same time. The median time from randomisation to scan was 2.7 weeks (interquartile range, IQR, 1.7-4.0). The uterus was reported to be present on the scan report in 38,221 and absent in 9,398 women. The latter included 3 women who had congenital uterine agenesis. In 593 women the ultrasound report was inconclusive with regard to presence or absence of uterus on this initial scan. Of these women, 242 had a TVS, 299 had a TA and 52 had both scans performed. As the 593 women went on to have 2 to 11 annual scans as part of the trial, these and all the other scans they have had were then queried to ascertain presence or absence of a uterus. On subsequent scans, in 353 presence of a uterus was recorded, 22 had the absence of a uterus clearly stated and 218 remained inconclusive due to difficulty

For peer review only - http://bmjopen.bmj.com/site/about/guidelines.xhtml

visualising the pelvis (Figure 1). Excluding the latter and women with congenital uterine agenesis, 47,994 women were therefore eligible for the analysis with ultrasound confirming presence of a uterus in 39,121 and absence of a uterus in 8,873.

Of the 47,994 women eligible for analysis, 8,871 self-reported hysterectomy at recruitment (RQ) and this was later confirmed on scan for 8,771 women. Of the 39,123 women who did not report hysterectomy on RQ, 39,021 had a uterus visualised at scan. In 102 women who did not report hysterectomy, a uterus was not found despite good visualisation of the pelvis.

At the interview (IF) prior to the scan, of the 47,994 women, the ultrasonographer recorded that 8,641 reported hysterectomy and 8,505 had it confirmed by scan data. Of the 39,353 women who did not report hysterectomy on IF, 38,985 had a uterus visualised at scan. Of the 8,871 women who self-reported hysterectomy on the RQ, 8,487 also reported it when interviewed immediately prior to the scan (IF) and 8457 had it confirmed by scan data. From the 39,123 women who did not report hysterectomy at RQ, 38,969 did not report it at IF either and the uterus was seen in 38,969. Seventy of the 384 women who self-reported hysterectomy on RQ but not on IF had a uterus visualised, whilst in 106 of the 154 women who did not report hysterectomy at RQ but did on IF the uterus was present. 54 women had uterine absence noted at the scan despite not reporting having had a hysterectomy at either the RQ or IF.

Performance characteristics

The validity, sensitivity, specificity, positive predictive value (PPV) and negative predictive value (NPV) of using RQ alone were 99.6%, 98.9%, 99.7%, 98.9% and 99.7%. Using the interview data alone, these were 98.9%, 98.4%, 99.1%, 95.9% and 99.7%. A McNemar test for paired proportions showed that RQ had greater validity than IF (99.6% versus 98.9%; $p<0.0001$). Consistent self-reporting at both RQ and IF resulted in validity, sensitivity, specificity, PPV and NPV of 99.8%, 99.6%, 99.9%, 99.4% and 99.9%, respectively (Table 2).

For peer review only - http://bmjopen.bmj.com/site/about/guidelines.xhtml

Table 2: Validity, sensitivity, specificity, PPV and NPV of self-reporting hysterectomy compared to the gold standard of ultrasound scanning

	**Self-reported hysterectomy**		
	**Recruitment Questionnaire (RQ)**	**Interview format (IF)**	**RQ and IF**
**Validity**	99.6%	98.9%	99.8%
**Sensitivity**	98.9%	98.4%	99.6%
**Specificity**	99.7%	99.1%	99.9%
**PPV**	98.9%	95.9%	99.4%
**NPV**	99.7%	99.7%	99.9%

Correlation with age and Index of Multiple Deprivation (IMD) score

The women who accurately reported the presence or absence of uterus (whether on RQ, IF or at both RQ and IF) were younger (median of 60.5, IQR 56.0-66.0) compared to those who were inaccurate in their reporting (median of 63.5, IQR 56.6-69.2) for RQ, 61.9 (IQR 56.5-67.7) for IF, 64.6 (IQR 57.9-69.1) for RQ and IF (Figure 2a). A Mann-Whitney test of the difference in rankings was significant at the 1% level (p=0.0006). The IMD score in those accurately reporting the presence or absence of a uterus was 15.0 (IQR 8.9-26.3) compared to 17.6 (IQR 10.9-29.4) for RQ, 16.4 (IQR 9.6-26.9) for IF and 18.2 (IQR 9.0-28.3) for both RQ and IF in those who were inaccurate (Figure 2b). A Mann-Whitney test showed some evidence of a difference in IMD score rankings (p=0.0214).

Discussion

To our knowledge, this is the largest study to date to assess the validity of self-reported hysterectomy by comparing it to a gold-standard of a pelvic ultrasound scan performed at a median of 2.7 weeks following questionnaire reporting. Self-reporting of hysterectomy was

For peer review only - http://bmjopen.bmj.com/site/about/guidelines.xhtml

correct in 99.6% of cases indicating that self-reporting (as captured in epidemiological studies) is an accurate measure of hysterectomy status. The validity of capturing hysterectomy using the questionnaire or interview was similar (99.6% versus 98.9%) though statistically significant. As the data on hysterectomy collected in most studies is usually ascertained through self-reporting, we feel that the data presented here can inform epidemiologists about the reliability of self-reporting of this variable.

The strengths of our study are (1) the size, (2) prospective design and (3) the fact that UKCTOCS participants were recruited from 13 centres across England, Wales and Northern Ireland through invitation from population registries – they are therefore as representative of the general female population aged 50-74 as is possible in clinical research.[14] We have previously observed a 'healthy- volunteer effect' in the trial[15] so the women participating in UKCTOCS may be more health-conscious and aware of medical procedures than the general population therefore being highly accurate in reporting. All women randomised to the ultrasound arm of UKCTOCS who attended for the initial scan were included in the analysis. The scans were in majority transvaginal, performed within 3 weeks of self-reporting with all ultrasonographers using similar ultrasound machines and following a uniform protocol and data capture form to record findings.[2] Where there was discrepancy or ultrasound records were inconclusive, all serial scans undertaken in the women during screening were analysed. Furthermore two types of self-reporting (questionnaire versus interview format) were assessed and their validity compared.

In our study, nearly 48,000 women were included in analysis with prospective evaluation of hysterectomy. Previous studies have been retrospective with medical note review used to confirm hysterectomy status. Logistical issues have meant that these studies were relatively small. They frequently encountered difficulties in acquiring notes because they relied upon patients remembering details of hospitals where a procedure, which potentially occurred decades ago, was undertaken and there was often poor recording of events.[9,10] In the multi-

For peer review only - http://bmjopen.bmj.com/site/about/guidelines.xhtml

centre Study of Women Across the Nation (SWAN), of the 3,302 participants, 239 reported hysterectomies of which for only 165 (69.3%) women the procedure could be confirmed. The remaining 74 could not be reviewed due to consent issues, loss to follow up or unavailability of medical records.[16] In addition some studies have used self-reported hysterectomy transcribed in medical histories as part of validation.[9] This has resulted in agreement of self-reported hysterectomy with medical records ranging from 66-99%.[9-13] A methodology that avoids several of these difficulties is to identify a cohort of women who have undergone hysterectomy using electronic outpatient records and subsequently investigate their self-reported hysterectomy status. Phipps and Buist, using this approach found that of 1,935 women with confirmed hysterectomy, 1,757 self-reported correctly, giving a sensitivity of 91%.[9]

Our study showed validity of self-reported hysterectomy of 99% in the UK women from the general population, aged 50-74. These figures are similar to the 100% accuracy rates reported in a subgroup of 200 women, for whom medical records could be obtained, participating in the Nurses' Study.[11] However this cohort was composed entirely of women with a nursing background who are well aware of the procedure, making it difficult to extrapolate to the wider population. Our results indicate that self-reporting of hysterectomy is highly accurate in women from the UK and we feel that our findings are applicable to women from other developed countries. However, it is possible that the validity of self-reporting of hysterectomy may be lower in those societies where there is a stigma attached to having undergone this procedure.

A limitation of our study is that the sonographer was not asked to specifically capture the presence or absence of a uterus. Endometrial thickness or endometrial abnormality were the only uterine details that were recorded.

For peer review only - http://bmjopen.bmj.com/site/about/guidelines.xhtml

A small proportion of women reported hysterectomy at recruitment but failed to do so at the scan. The reason for this discrepancy may be that either the sonographer did not question the woman or the woman did not hear the question at the time of the scan.

The women who were accurate in their reporting were younger than those that were inaccurate in their reporting of presence or absence of uterus. One of the reasons for this may be due to the procedure being done many years, even decades previously.

In our study 1.2% of women (576 of 47,994) were inconsistent in their self-reporting of hysterectomy status between the recruitment questionnaire and the interview carried out at a median 2.7 weeks later. Studies examining agreement of self-reports report a similarly low rate of inconsistency but these were made several years apart.[17,18] One of the reasons for this may be that the women were more accurate in reporting on the questionnaire as they had more time to fill this in compared to those attending their first scan in a busy clinic. Our and previous data indicate that capturing data via interviewer-administered questionnaire is a valid method to capture self-reported medical history.

Conclusion

Our data indicates that self-reported hysterectomy, whether through questionnaire or interview, is highly accurate and is a reliable data source for studying long term associations of hysterectomy with disease onset.

For peer review only - http://bmjopen.bmj.com/site/about/guidelines.xhtml

For peer review only

Acknowledgements

We are particularly grateful to the women throughout the UK who are participating in the trial and to the entire medical, nursing, and administrative staff who work on UKCTOCS.

Contributors: UM and AGM were involved in study design and concept and drafted the manuscript. UM, AGM and HT did the literature search for this manuscript and prepared the tables. UM, AGM and MB did the statistical analysis. JK, AR, AS, SA, SC and IJ were involved in the interpretation of the data. All authors critically revised the manuscript and approved the final version. UM is the guarantor.

Funding: UKCTOCS was core funded by the Medical Research Council, Cancer Research UK, and the Department of Health with additional support from the Eve Appeal, Special Trustees of Bart's and the London, and Special Trustees of UCLH and supported by researchers at the National Institute for Health Research University College London Hospitals Biomedical Research Centre. The funding source or the sponsor had no role in data collection, data analysis, data interpretation, or writing of the report. The researchers are independent from the funders.

Competing interests: IJ has a consultancy arrangement with Becton Dickinson in the field of tumour markers and ovarian cancer, UM has a financial interest through UCL Business and Abcodia Ltd in the third party exploitation of clinical trials biobanks which have been developed through the research at UCL. None of the other authors have any conflict of interest; no other relationships or activities that could appear to have influenced the submitted work.

Patient consent: Obtained.

For peer review only - http://bmjopen.bmj.com/site/about/guidelines.xhtml

For peer review only

Ethics approval: The study was approved by the UK North West Multicentre Research Ethics Committees (North West MREC 00/8/34) with site specific approval from the local regional ethics committees and the Caldicott guardians (data controllers) of the primary care trusts.

Data sharing statement: No additional data are available.

For peer review only

For peer review only - http://bmjopen.bmj.com/site/about/guidelines.xhtml

References

1. Johnson N, Barlow D, Lethaby A, Tavender E, Curr E, Garry R. Surgical approach to hysterectomy for benign gynaecological disease. *The Cochrane database of systematic reviews.* 2006(2):CD003677.
2. Menon U, Gentry-Maharaj A, Hallett R, et al. Sensitivity and specificity of multimodal and ultrasound screening for ovarian cancer, and stage distribution of detected cancers: results of the prevalence screen of the UK Collaborative Trial of Ovarian Cancer Screening (UKCTOCS). *The lancet oncology.* Apr 2009;10(4):327-340.
3. The Million Women Study: design and characteristics of the study population. The Million Women Study Collaborative Group. *Breast cancer research : BCR.* 1999;1(1):73-80.
4. Garry R. The future of hysterectomy. *BJOG : an international journal of obstetrics and gynaecology.* Feb 2005;112(2):133-139.
5. Merrill RM, Layman AB, Oderda G, Asche C. Risk estimates of hysterectomy and selected conditions commonly treated with hysterectomy. *Annals of epidemiology.* Mar 2008;18(3):253-260.
6. Hankinson SE, Hunter DJ, Colditz GA, et al. Tubal ligation, hysterectomy, and risk of ovarian cancer. A prospective study. *JAMA : the journal of the American Medical Association.* Dec 15 1993;270(23):2813-2818.
7. Ingelsson E, Lundholm C, Johansson AL, Altman D. Hysterectomy and risk of cardiovascular disease: a population-based cohort study. *European heart journal.* Mar 2011;32(6):745-750.
8. Press DJ, Sullivan-Halley J, Ursin G, et al. Breast cancer risk and ovariectomy, hysterectomy, and tubal sterilization in the women's contraceptive and reproductive experiences study. *American journal of epidemiology.* Jan 1 2011;173(1):38-47.
9. Phipps AI, Buist DS. Validation of self-reported history of hysterectomy and oophorectomy among women in an integrated group practice setting. *Menopause.* May-Jun 2009;16(3):576-581.
10. Green A, Purdie D, Green L, Dick ML, Bain C, Siskind V. Validity of self-reported hysterectomy and tubal sterilisation. The Survey of Women's Health Study Group. *Australian and New Zealand journal of public health.* Jun 1997;21(3):337-340.
11. Colditz GA, Stampfer MJ, Willett WC, et al. Reproducibility and validity of self-reported menopausal status in a prospective cohort study. *American journal of epidemiology.* Aug 1987;126(2):319-325.
12. Brett KM, Madans JH. Hysterectomy use: the correspondence between self-reports and hospital records. *American journal of public health.* Oct 1994;84(10):1653-1655.
13. Horwitz RI. Comparison of epidemiologic data from multiple sources. *Journal of chronic diseases.* 1986;39(11):889-896.
14. Menon U, Gentry-Maharaj A, Ryan A, et al. Recruitment to multicentre trials--lessons from UKCTOCS: descriptive study. *Bmj.* 2008;337:a2079.
15. Burnell M, Gentry-Maharaj A, Ryan A, et al. Impact on mortality and cancer incidence rates of using random invitation from population registers for recruitment to trials. *Trials.* 2011;12:61.
16. Morelli SS, Lian Y, Schott LL, Weiss G. Qualifications of Physicians Performing Hysterectomy: The Study of Women's Health Across the Nation. *Reproductive sciences.* Dec 10 2009.
17. Lucas R, Azevedo A, Barros H. Self-reported data on reproductive variables were reliable among postmenopausal women. *Journal of clinical epidemiology.* Sep 2008;61(9):945-950.
18. Bosetti C, Tavani A, Negri E, Trichopoulos D, La Vecchia C. Reliability of data on medical conditions, menstrual and reproductive history provided by hospital controls. *Journal of clinical epidemiology.* Sep 2001;54(9):902-906.

For peer review only - http://bmjopen.bmj.com/site/about/guidelines.xhtml

Figure legends

Figure 1: UKCTOCS participants randomised to the ultrasound group who underwent the initial scan on the trial which confirmed presence or absence of a uterus

Figure 2: **(a)** Kernel density plots of age at randomisation and **(b)** Index of Multiple Deprivation (IMD) for those that were accurate in their reporting versus those that were inaccurate

For peer review only

For peer review only - http://bmjopen.bmj.com/site/about/guidelines.xhtml

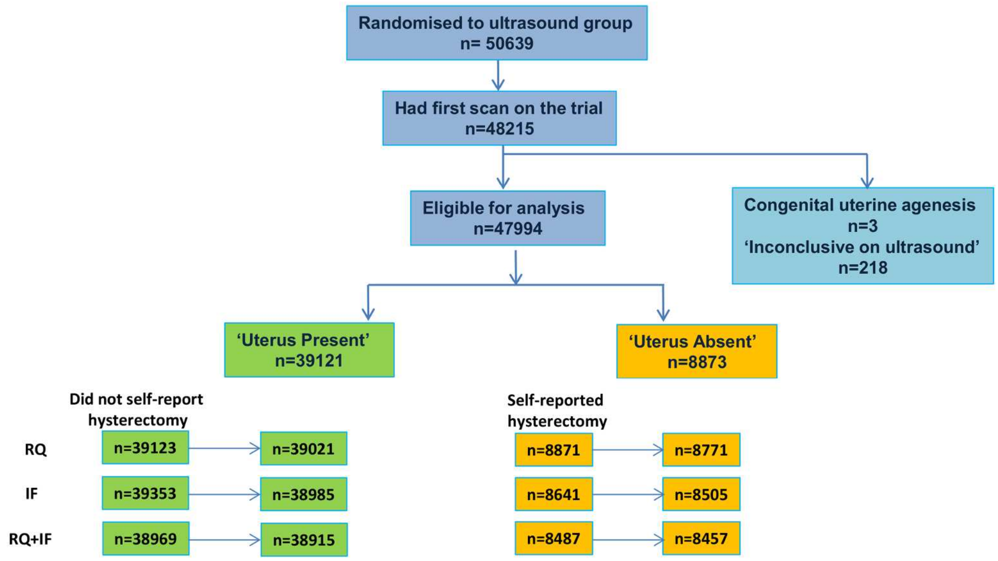

UKCTOCS participants randomised to the ultrasound group who underwent the initial scan on the trial which confirmed presence or absence of a uterus
173x100mm (300 x 300 DPI)

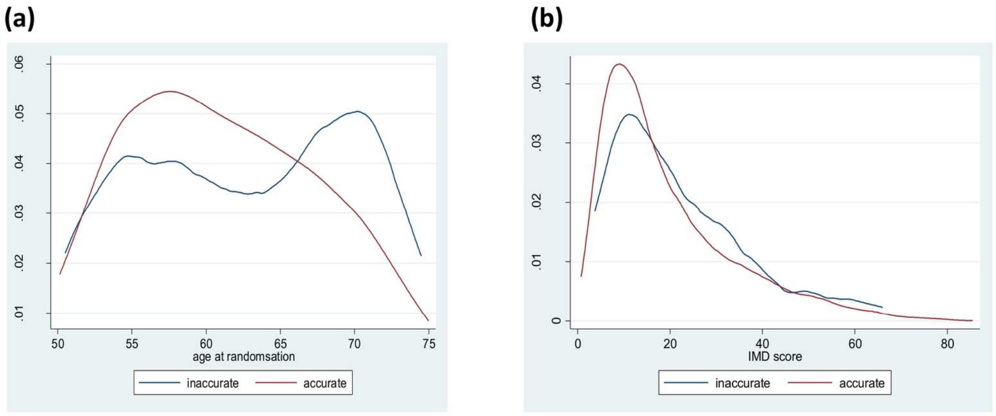

(a) Kernel density plots of age at randomisation and (b) Index of Multiple Deprivation (IMD) for those that were accurate in their reporting versus those that were inaccurate
173x73mm (300 x 300 DPI)

STROBE Statement		
**Item**	**Item No**	**Pages**
**Title and Abstract**	1	Pages 1-3
**Introduction**		
Background/rationale	2	Pages 5-6
Objectives	3	Page 6
**Methods**		
Study design	4	Pages 6-7
Setting	5	Pages 6-7
Participants	6	Pages 6-7
Variables	7	Pages 7-8
Data sources/measurement	8	Pages 7-8
Bias	9	Pages 7-8
Study size	10	Pages 6-8
Quantitative variables	11	Pages 7-8
Statistical methods	12	Pages 7-8
**Results**		
Participants	13	Pages 8-10
Descriptive data	14	Pages 8-10
Outcome data	15	Pages 8-10
Main results	16	Pages 8-10
Other analyses	17	Pages 8-10
**Discussion**		
Key results	18	Page 10-11
Limitations	19	Pages 12
Interpretation	20	Pages 11-12
Generalisability	21	Page 11-12
**Other information**		
Funding	22	Page 14

For peer review only
